# The many “faces” of copper in medicine and treatment

**DOI:** 10.1007/s10534-014-9736-5

**Published:** 2014-04-20

**Authors:** Anna Hordyjewska, Łukasz Popiołek, Joanna Kocot

**Affiliations:** 1 Chair and Department of Medical Chemistry, Faculty of Medicine, Medical University of Lublin, 4A Chodzki Street, 20-093 Lublin, Poland; 2 Chair and Department of Organic Chemistry, Faculty of Pharmacy, Medical University of Lublin, 4A Chodzki Street, 20-093 Lublin, Poland

**Keywords:** Copper, Wilson’s disease, Menke’s disease, Enzymes, Alzheimer’s disease, Metallothionein, Aceruloplasmin, Copper complexes

## Abstract

Copper (Cu) is an essential microelement found in all living organisms with the unique ability to adopt two different redox states—in the oxidized (Cu^2+^) and reduced (Cu^+^). It is required for survival and serves as an important catalytic cofactor in redox chemistry for proteins that carry out fundamental biological functions, important in growth and development. The deficit of copper can result in impaired energy production, abnormal glucose and cholesterol metabolism, increased oxidative damage, increased tissue iron (Fe) accrual, altered structure and function of circulating blood and immune cells, abnormal neuropeptides synthesis and processing, aberrant cardiac electrophysiology, impaired myocardial contractility, and persistent effects on the neurobehavioral and the immune system. Increased copper level has been found in several disorders like e.g.: Wilson’s disease or Menke’s disease. New findings with the great potential for impact in medicine include the use of copper-lowering therapy for antiangiogenesis, antifibrotic and anti-inflammatory purposes. The role of copper in formation of amyloid plaques in Alzheimer’s disease, and successful treatment of this disorder in rodent model by copper chelating are also of interest. In this work we will try to describe essential aspects of copper in chosen diseases. We will represent the evidence available on adverse effect derived from copper deficiency and copper excess. We will try to review also the copper biomarkers (chosen enzymes) that help reflect the level of copper in the body.

## Distribution, storage, uptake

Copper is absorbed from diet, mainly in duodenum, although it is thought that some absorption takes place in the stomach and in the distal part of the small intestine. Copper uptake into enterocyte is managed mainly by human copper transport protein 1 (hCTR1). The processes is mediated by action of reductases in the apical membrane, which reduce dietary Cu(II) to Cu(I)—the acceptable state of copper for this receptor. Recent studies show that transport of Cu(I) ions can be carried out using the divalent metal transporter 1—it is an iron transport protein located on the membrane of enterocytes. Following absorption by the gut, copper is secreted into the circulation and bound as Cu(II) to albumin, transcuprein and low molecular weight copper-histidine complexes. Upon gaining the liver, copper is rapidly taken up by hepatocytes also via hCTR1. Inside the cytoplasm of cells, copper is either chelated by metallothionein (MT) for storage or it binds to a copper chaperone for delivery to specific proteins or it binds to reduced glutathione. Three copper chaperones have been identified: CCS (copper chaperone for superoxide dismutase 1), COX17 (chaperone for cytochrome c oxygenase) and ATOX1 (chaperone for ATPases—ATP7A and B). ATP7A delivers copper to tyrosinase and ATP7B to lysyl oxidase and ceruloplasmin (Cp) (Turecky et al. 1984; de Romana et al. 2011).

Multicompartment studies with isotopic traces and kinetic modeling confirm that there is a relatively little copper storage, about 80–100 mg, mostly in the liver, followed by brain, kidney and heart (Jacobs and Wood 2003; Thiele 2003; Peña et al. 2000). Copper is excreted into gastrointestinal tract, either via the bile or as non-absorbed copper. Only 10–15 % of copper in bile is reabsorbed. Excess of copper is eliminated in feces, both as absorbed and unabsorbed metal ions and from biliary excretion, averaging 0.5–1.3 mg per day, and with small amounts in urine, saliva and perspiration. The average intake of copper by human adults varies from 0.6 to 1.6 mg per day and the main sources are seeds, grains, nuts and beans, shellfish and liver (Tapiero et al. 2003; Thiele 2003). Several dietary factors have been proposed as possible modifiers of copper absorption in humans. For example recent studies have shown that patients who have used the denture cream enriched with zinc too often, suffered from progressive myelopolyneuropathy which was due to copper deficiency (Hedera et al. 2009; Nations et al. 2008; de Romana et al. 2011). Certain prebiotic, naturally present or added to food, such as short-chain fructooligosaccharides, pectin and inulin, have a positive effect on copper absorption. Ascorbic acid and its salts used in food industry (E300—E3004), phytate (found in cereals—especially bran, seeds of legumes and nut), EDTA-Na/Fe and polyphenols do not seem to affect its absorption.

## Diseases related with copper deficiency or excess

### Menke disease and Wilson disease

Essentiality and toxicity of copper are well characterized by two rare genetic conditions: Menkes disease (MD) and Wilson disease (WD). Wilson’s disease (autosomal recessive) and Menke’s disease (X-linked) represent the most well recognized and understood disorders of copper homeostasis (Shim and Harris 2003). They are caused by recessive defects in genes, ATP7B and ATP7A, which are differentially expressed in tissues (ATP7B is expressed in the TGN membrane of hepatocyte, ATP7A-on the TGN membrane of the placenta, gut and brain respectively). Both the proteins made by ATP7A and by ATP7B are membrane-bound copper-transporting ATPases (Shim and Harris 2003).

Menke disease is an X-linked inherited disorder, and it is caused by a mutation in the ATP7A gene. This gene encodes a copper transporting ATPase. The ATP7A gene product functions as an intracellular pump to transport copper into the trans-Golgi network for incorporation into copper requiring enzymes including dopamine β–hydroxylase and also mediates copper exodus from cells. Mutations in this gene appear as disorders of copper deficiency and lead to hypothermia neuronal degeneration, mental retardation abnormalities in hair (scalp hair called kinky or steel wool), bone fractures, and aortic aneurysms (Uriu-Adams and Keen 2005). A failure of function of ATP7A in the intestine leads to a failure of copper efflux from intestine, accumulation of excess copper in the intestine, a failure of copper absorption into the blood, and generalized copper deficiency. The failure of function of ATP7A in the blood brain barrier leads to a failure of copper efflux from cells of this barrier, accumulation of copper in these cells, and a failure of copper uptake in the brain, even if circulating copper levels are normalized by parenteral copper therapy (Brewer 2003a, b).

Wilson’s disease (WD) is an autosomal recessive genetic disorder, and is caused by disabling mutations in both copies of the ATP7B gene. This gene is responsible for copper’s transfer into the secretory pathway for both binding into Cp and excretion into the bile (Brewer 2000). Clinical manifestations are liver disease and neurological damage. The cornea of the eye is also affected, resulting in the hallmark brown discoloration of the cornea, which is very specific for neurological WD, the Kayser Fleischer ring” (Tapiero et al. 2003). Despite high hepatic copper levels, Cp and copper concentrations in blood are low, while urinary copper excretion is increased. When Wilson’s disease is recognized in time, it can be treated in several ways, including the use of chelating agents (such as EDTA-K_2_/Ca, 2,3-dimercaptopropan-1-ol), high levels of zinc supplements (40–50 mg three times per day) and low Cu diet (Uriu-Adams and Keen 2005). The therapy of Wilson’s disease is mostly based on Zn addition to the food, penicillamine addition and tetrathiomolybdate (TM) or trientine (triethylene tetramine dihydrochloride). The first line therapy for asymptomatic patients and for those with mild hepatic disease is zinc in form of sulphate or acetate. Main mechanism in which zinc is efficacious for Wilson disease is that zinc competes with copper for similar binding sites—its mechanism of action depends on interfering with uptake of copper from the gastrointestinal tract (Abuduxikuer and Wang 2014; Wang et al. 2013; Wiernicka et al. 2013; Ugarte et al. 2013). Zinc probably may also act by induction of intestinal cell metallothionein. Metallothionein T, once induced, has a high affinity for binding copper and prevents the serosal transfer of copper into the blood. The intestinal cells turnover rapidly and take the complexed copper into the stool for final excretion. Zinc not only blocks food copper but also the copper that is endogenously excreted via salivary, gastric, and other gastrointestinal juices. Thus, zinc is effective in producing a chronic negative copper balance (Litwin et al. 2013; Prasad 2013; Roney and Colman 2004; Ugarte et al. 2013). Penicillamine mechanism of action involves reductive chelation of copper in the body, and enhanced excretion of copper in the urine (Butler et al. 2001; Brewer 2000). Penicillamine binds copper with sulfhydryl groups and this complex is subsequently removed from the organism via the urine. Trientine has a polyamine-like structure that allows copper binding by forming a stable complex with four constituent nitrogen’s in a planar ring. Like penicillamine, trientine promotes copper excretion in the urine (Gromadzka et al. 2014). TM mechanism of action involves the formation of a tripartite complex with protein and copper, and in this way prevents the absorption of copper from gastrointestinal tract. TM taken with meals associates with copper present in food, thereby preventing copper reabsorption (Yoshii et al. 2001). If TM is given between meals, it is also absorbed into the bloodstream, where it binds either free copper ions or copper loosely bound with serum albumin (Brewer et al. 2000). Such complex is no longer available for cellular uptake, possesses no biological activity and is cleared in bile and urine (Kalita et al. 2014; Nasulewicz et al. 2004).

### Infantile and childhood copper toxicosis syndromes

Syndrome called idiopathic copper toxicosis (ICT) occurring in infants and young children has been seen in many parts of the world, and is usually associated with high levels of hepatic copper, caused by high concentrations of copper in drinking water or food (Brewer 2003a, b). In west Austria, Germany and in Italy in years 1900–1980 about 138 infants died because of this disease. Similarly, in India the infants who were fed milk stored in brass or copper containers developed a disease known as Indian Childhood Cirrhosis (ICC), in which liver has increased levels of copper. ICC is characterized pathologically by a micro-micronodular cirrhosis, accumulation of Mallory’s hyaline in necrotic hepatocytes, distinctive orcein positive granules and striking lack of regenerative nodule, with no chronic biliary disease (Baker et al. 1995). All these syndromes (ICT and ICC) are familiar. It has been suggested that these disorders result from Wilson’s disease gene, well established as causing mild copper accumulation, but causing no medical problems with the usual intake of copper. The concept is that when the main source of nutrition and/or water for infants has a high content of copper, Wilson’s disease can become a dominant, and manifests in heterozygote’s (Butler et al. 2001). An alternate hypothesis is that there is a gene, not discovered yet, which impairs copper excretion causing these syndromes (Brewer 2003a, b).

### Aceruloplasminemia

Aceruloplasminemia—an autosomal recessive disease—is caused by mutations in the Cp allele on chromosome 3q, and results in a total absence of Cp in the blood. Therefore an iron accumulation, causing clinical problems in the brain and liver is observed (Brewer 2003a, b). The absence of Cp does not produce marked changes in copper metabolism but only alters iron metabolism. It produces a gradual accumulation of iron in the liver, pancreas, retina and central nervous system. Cp possesses ferroxidase activity for mobilization and utilization of the stored iron from Fe(II) to Fe(III) (Tapiero et al. 2003; Harris et al. 1998), but it isn’t the only way of in which iron ions, could be oxidized. They can be oxidized for transferrin loading also by hephaestin, cytochromes and in Fenton—like- reactions (Chen et al. 2014).

### Alzheimer disease

Alzheimer disease (AD) is a multifactorial disorder in which many altered cellular processes are involved, such as oxidative stress, neuroinflammation, impairments in energy metabolism, and others. Major histopathological features associated with AD are senile plaques and neurofibrillary tangles, related to aberrant processing of amyloid precursor protein (APP) leading to the deposition of amyloid β and hyperphosphorylation of tau protein, respectively (Gonzalez-Dominguezet al. 2014, Arnal et al. 2013; Atwood et al. 2000). The main brain regions affected in AD include the entorhinal cortex, hippocampus, basal forebrain and amygdale, which exhibit synaptic loss resulting in extensive brain atrophy. Clinically, AD patients present with symptoms of memory loss, altered personality and behavior, and impaired executive function. The estimated copper content in a healthy human adult brain is 7–10 % of total body copper, similar to that found in the liver, the major organ of copper homeostasis. In a human adult brain, copper is particularly enriched in the hippocampus and substantia nigra (Hung et al. 2013).

Recent findings report that alteration of homeostasis of biological metallic ions including zinc, iron, aluminum and copper, plays an important role in the pathogenesis of AD. Special attention has been paid on homeostasis of copper (Duce et al. 2010; Uriu-Adams and Keen 2005; White et al. 1999; Ugarte et al. 2013). Copper is an important transition metal in various metabolic pathways and prevents excessive activity of *N*-methyl-d-aspartate receptors, which are excitatory neurotransmitter receptors (Noda et al. 2013; Eskici and Axelsen 2012). Copper levels in plasma and free serum copper levels are known to elevate with aging and to be elevated in AD brains (Noda et al. 2013; Eskici and Axelsen 2012). Some authors demonstrated that free copper (also known as NCBC or non-ceruloplasmin-bound Cu) is elevated in the blood of AD patients, negatively correlates with cognition, and predicts the rate of loss of cognition (Arnal et al. 2013). Other studies suggest that copper can promote amyloid β aggregation. In addition, unusually high concentration of copper (400 μM) has been observed in Alzheimer’s senile plaques. A number of reports have hypothesized that homodimerization of APP can affect amyloid β production. Interestingly, some copper binding domains have been identified on APP, next to the growth factor-like domain within the N-terminal domain that consists of four amino acids, His-147, His-151, Try-168 and Met-170 (Noda et al. 2013; Eskici and Axelsen 2012; Atwood et al. 2000).

Metal-induced oxidative stress is another mechanism that can lead to profound neurodegenerative processes in AD. As copper is a redox active metal, aberrations in its’ homeostasis may create favorable conditions for superoxide-yielding redox cycling and oxidative damage to susceptible regions in brain, where easily oxidized substrates abound (e.g. membrane polyunsaturated lipids), and redox-based neurochemical reactions occur (Gromadzka et al. 2014, Lopes da Silvaet al. 2013). Paradoxically, the utilization of copper’s redox activity in Fenton or Haber–Weiss reactions promotes the production of toxic reactive oxygen species (ROS). Therefore, the dichotomous nature of copper demands a precise regulation to maintain an appropriate level and distribution in the brain, and to prevent inadvertent interactions with other cellular components (Hung et al. 2013; Duce et al. 2010).

### Prion diseases

The cellular prion protein, PrPC, a copper binding protein located predominantly at the synapse, is GPI-anchored cell surface glycoprotein, found in the brain, spinal cord and peripheral tissues. It has a causative role in the pathogenesis of Creutzfeldt–Jacob disease, Gerstmann–Straussler–Scheinker disease and fatal familial insomnia, known as prion diseases (Mittergger et al. 2009; Hornshaw et al. 1995a, b). PrPC is reported to have copper-dependent SOD-like activity. It is a 33-35 kDa protein with four or five ions of Cu bound to four identical sequences of eight amino acids in the N-terminal region of the protein. Copper lends structural stability to the N-terminal region and also to other parts of the molecule (Tapiero et al. 2003; Hornshaw et al. 1995a, b). Transition of PrPC from a predominantly α-helical form to a β-sheet rich isoform seems to be the cause of transmissibility and pathogenesis (Brown 2003; Kramer et al. 2001). In the late 90’s, it was shown that copper stimulates endocytosis of the prion protein. Copper stimulation of PrPC endocytosis requires the highly conserved Cu(II) binding repeats in the protein (Mittergger et al. 2009; Hijazi et al. 2003; Pauly and Harris 1998). It was also shown that copper ions facilitate the renaturation of guanidine-denatured PrPC molecules to form the protease-resistant infectious prion particle that accumulates in the endosome due to defective recycling of this particle to the plasma membrane. It has been reported that the enhanced expression of PrPC increases cell stability to take up copper (Pauly and Harris 1998; Ironside 1996). It also appears to increase its resistance to copper toxicity and oxidative stress. Exposure on to large non-physiological concentrations of Cu(II) ions induces endocytosis of this protein and brings its copper into the cell. It seems that the role of Cu(II) ions in the pathogenesis of prion disease is rather complex (Tapiero et al. 2003; Bocharova et al. 2005). Copper may change the structure and convert capability of PrPC with further implications to cell signaling (Brown 2003; Hijazi et al. 2003). But on the other hand a higher availability of copper may induce some kind of oxidative stress conditions that generate an increased level of PrPC (Kramer et al. 2001).

### Diabetes

Diabetes mellitus is often found in aceruloplasminemic individuals. It is currently thought that the diabetes is an adverse consequence of increased Fe ions accumulation in the pancreas due to the lack of ferroxidase activity in the tissue (Uriu-Adams and Keen 2005). While altered copper metabolism can directly or indirectly alter glucose homeostasis, conversely, diabetes can perturb copper metabolism (Takahashi et al. 1996). In rodents, diabetes results in increased copper concentration in the liver and kidney, which is associated with an increased metallothionein level. The presence of copper ions in blood can increase the rate of advanced glycosylated end products formation, which is associated with the pathogenesis of secondary complications in diabetes. Others studies have shown that plasma concentrations seemed to be higher in diabetic humans, compared to non-diabetic—plasma copper was particularly high in diabetics with complications, such as retinopathy, hypertension and microvascular disease (Uriu-Adams and Keen 2005).

### Angiogenesis and cancer

The role of copper in the growth and progression of malignancy has been the subject of intense investigations, since copper ions generate free radicals, which may activate signaling pathways for cell proliferation. The evidence obtained over the years has shown that cancer cells generally require more copper for their growth and metabolism than normal resting cells. Therefore agents that affect copper homeostasis are of interest for cancer therapy (Easmon 2002). High serum and tissue levels of copper were found in various human tumors including: Hodgkin’s lymphoma, leukemia, sarcoma, brain, cervix, lung, breast and liver cancers. It has also been shown that serum copper levels return to normal on remission of the disease or upon removal of the tumor. It has been demonstrated that copper is required for angiogenic process (Nasulewicz et al. 2004; Brewer et al. 2000; Turecky et al. 1984). Many angiogenic promoters also appear to be dependent on normal level of copper. Angiogenesis is a complex process that is essential for the tumor growth, invasion and metastasis. In vitro studies have shown that copper acts as a critical angiogenic effector by stimulating the proliferation and migration of endothelial cells. Recent studies provide evidence that limiting the biological availability of copper by penicillamine or TM administration slows tumor growth (Tapiero et al. 2003). Lowering copper level moderately with a drug such as TM produces strong antiangiogenesis and potent anticancer effects in several rodent models (Cox et al. 2001). There may be multiple mechanisms accounting for the effect of copper-lowering therapy by TM to inhibit tumor angiogenesis. But it is most probable that TM suppresses NF-κB protein levels and transcription, and NF-κB is known as regulator of many genes involved in tumor invasion, angiogenesis and metastasis (Pan et al. 2002). Recent studies have shown that copper directly stimulates angiogenesis. Others studies have shown that copper seems to be necessary for endothelial cell activation, as it stimulates their proliferation and migration (Nasulewicz et al. 2004). Several angiogenic factors, such as VEGF, basic fibroblast growth factor, tumor necrosis factor alpha and interleukin 1 have been found to be copper activated (Brewer et al. 2000). Activation factors bind to endothelial cells, switch them from G0 into G1 phase and force proliferation (Brewer 2003a, b; Yoshiii et al. 2001). Copper also binds to several proteins (heparin, ceruloplasmin), which therefore gain angiogenic activity manifested by stimulation of endothelial cells (Turecky et al. 1984). Mild copper deficiency in animals strongly inhibits the response to various normally angiogenic stimuli when placed in the cornea. It has been showed that tumors implanted in the brains of copper-deficient animals had reduced growth and invasive properties (Pan et al. 2002; Cox et al. 2001).

### Immune response

Most recently it has been discovered that fibrotic and inflammatory cytokines are also copper dependent. Copper metabolism is altered in inflammation process—its level in serum rises. Copper itself is important for immune response, including the production of IL-2 by activated lymphocytic cells and it supports the activity and effectiveness of cellular and humoral immunity by influence on T lymphocytes (Tapiero et. al 2003; Percival 1998).

### Cardiovascular disease

It has been demonstrated that experimental copper deficiency significantly increases susceptibility of lipoproteins and cardiovascular tissues to lipid peroxidation, thus increasing the risk of cardiovascular disease (Percival 1998). The heart and heart vessels are particularly vulnerable to copper deficiency. Alterations in cardiac morphology include enlarged myocytes, derangement of myofibrils, fragmented basal laminae at capillary-myocyte interfaces, and mitochondrial proliferation, swelling and fragmentation (Uriu-Adams and Keen 2005). Also electrocardiographic abnormalities and impaired contractile and mitochondrial respiratory functions are observed in copper deficient hearts. Hypertrophic cardiomyopathy is noted in humans with mutations in copper chaperone for cytochrome c oxidase. Copper deficiency leads also to anemia, since cytochrome oxidase is required for blood formation. Copper depletion experiments also show abnormalities in blood pressure and aortic stenosis (Uriu-Adams and Keen 2005; Rowland and Schneider 2001). Repletion of Cu by dietary copper supplementation reverses many of the adverse histopathological and hemodynamic effects on the heart, possibly through the normalization of the expression of genes, which are involved in contractility, calcium cycling, inflammation and extracellular matrix metabolism (Uriu-Adams and Keen 2005). There are also data showing a correlation between copper deficiency and atherosclerosis. An increased concentrations of total cholesterol and LDL with reduction of HDL are observed in the subjects fed an experimental diet low in copper (Brewer 2003a, b, Rowland and Schneider 2001).

### Oxidative stress related disorders

The occurrence of copper on two oxidize states allows it to serve as cofactor in redox reactions such as cytochrome c oxidase (involved in the mitochondrial electron transport chain) and superoxide dismutase (involved in the detoxification of reactive oxygen species). An excess of copper could result in oxidative stress related health disorders, many of which can be linked partially to its redox reactivity. Copper has been suggested to facilitate oxidative tissue injury through a free radical mediated pathway analogous to the Fenton reaction. Copper deficiency also affects directly or indirectly the components of the oxidant defense system and as a result increased ROS and oxidative damage to lipid, DNA and proteins have been observed in human cell culture models. Copper on Cu(I) state can bind directly to free thiols of cysteine or methionine, resulting in oxidation and subsequent cross-links between proteins leading to impaired activity. Copper on Cu(II) state prefers nitrogen donors, such as histidine, or oxygen donors, such as glutamate or aspartate.

## Temporary deficiency and toxicity of copper

Copper deficiency has been described mostly in infants on total parenteral nutrition without adequate mineral supplementation or in persons with persistent nephritic syndrome that increases copper losses. Low copper status has been associated with bone malformation during development, increased osteoporosis, impaired melanin synthesis, poor immune response, therefore increasing frequency of infections, cardiovascular disease, alterations with cholesterol metabolism, and disturbance of metabolism of other trace elements, such as iron mobilization.

Copper toxicity is usually an acute episode resulting from copper contamination of drinking water or food (Puig and Thiele 2002). The symptoms are nausea, vomiting and diarrhea, abdominal pain, headache, tachycardia, respiratory difficulties, hemolytic anemia. They begin in the most sensitive subjects at the concentration of 3 mg/l (Peña et al. 2000). Some investigators have suggested that chronic consumption of drinking water with elevated Cu concentration may be risky for susceptible populations, including infants, young children and individuals who are heterozygotic for Wilson’s disease (Uriu-Adams and Keen 2005; Ugarte et al. 2013).

## Cu-requiring/depending proteins

Copper is an important micronutrient that forms part of several proteins involved in a variety of biological processes indispensable to sustain life. Cu-requiring proteins are involved in a variety of biological processes and deficiency in these enzymes or alterations in their activities often cause disease states or pathophysiological conditions (Puig and Thiele 2002). Some authors consider these proteins as better indicators of metabolically active copper stores than the serum concentrations of copper, or Cp because the enzyme activities are sensitive to changes in copper stores.

### Ceruloplasmin (Cp)

Cp is a single polypeptide chain at about 132 kDa. The Cp protein contains seven copper ions, accounting for more than 70 % of the total copper circulating in the serum and it provides a major link between copper and iron metabolism (Harris et al. 1998). Besides its potential role in copper delivery to cells and excretion of copper from body, this glycoprotein presents ferroxidase activity and catalyzes the conversation of ferrous to ferric iron which is then transferred to transferrin. This change is helpful to provide iron, in the form needed to bind to transferrin (iron Fe(III) plasma carrier), as it emerges from cells for further transport from the bone marrow to red blood cells, where most iron resides (Harris et al. 1998). In severe copper deficiency, there is little or no copper containing Cp in plasma and tissues, and therefore, iron accumulation in the liver occurs (Tapiero et al. 2003; Turecky et al. 1984; Takahashi et al. 1996). Immunoreactive or enzymatically measured Cp levels are used to evaluate the body storage of copper, because Cp acts as an acute-phase reactive protein to stress and trauma conditions—its concentration is elevated in response to inflammation, infection and various chronic diseases such as arthritis.

### Cytochrome-c oxidase

Cytochrome c oxidase (CCO) is bound with inner mitochondrial membrane. It is a component of the mitochondrial respiratory chain so it takes part in energy production. It has four redox active metal sites, two hem sites and two copper sites. It is the terminal oxidase in most aerobic organisms and reduces molecular oxygen (O_2_) to water. In addition to O_2_ reduction, cytochrome C oxidase pumps protons from the inside to the outside of the membrane (Tapiero et al. 2003). Studies have shown that the activities of CCO were reduced in response to copper depletion.

### Metallothioneins (MTs)

Metallothioneins (MTs) are cysteine-rich metal binding proteins at low molecular weight. They come at least in two isoforms encoded by several genes. The biological roles of metallothioneins are: detoxification of metals ions, including non-essential and excess of essential metals ions, storage of essential microelements, sequestration of ROS and nitrogen species (Ogra et al. 2006). MTs bind Zn, Cu and Cd ions, mainly, however, copper is bound most tightly and can displace others ions. Under reducing conditions, metallothioneins can bind copper ions and render them redox inactive. Metallothioneins are induced under Cu-deficient conditions to maintain the activities of intracellular Cu-proenzymes such as cytochrome C oxygenase, and to scavenge ROS instead of copper containing antioxidants, such as Cu/Zn—SOD (Ogra et al. 2006).

### Cu/Zn superoxide dismutase

Cu/Zn—SOD converts superoxide anions to peroxide for further disposal (by catalase and glutathione peroxidase) and is found largely in cytosol. Mutations of Cu/Zn-SOD have been recently of interest in connection with amyotrophic lateral sclerosis, where a gain of function is responsible for the underlying neurological symptomatology (Roney and Colman 2004; Tapiero et al. 2003). The loss of kinetic stability is responsible for the exposition of the sulfide bond to reduction and hydrophobic surface area to potential interaction with other proteins (Itoh et al. 2009). Thereby, it has been suggested that the mutant protein binds copper abnormally, generating ROS. Copper is more important than zinc for kinetically stabilizing SOD. The absence of Cu from SOD may result in a protein prone to aggregation, possessing enhanced oxidative activity and abnormally interacting with other proteins (Lynch and Colón 2005)

## Enzymes activated by copper ions

### Catalase

Catalase is a Fe-binding enzyme that catalyzes the conversion of hydrogen peroxide to water and oxygen. Expression of cardiac-specific catalase prevents injury to the heart following oxidative insults. Copper deficiency can lead to a reduction in catalase activity in tissues, such as heart and liver (Uriu-Adams and Keen 2005).

### Glutathione peroxidase (GPx)

Glutathione peroxidase converts hydroperoxide compounds to hydroxide compounds. The activity of this enzyme has been reported to be decreased in case of copper deficiency in the liver and plasma. Cu deficiency may reduce GPx activity by decreasing enzyme’s mRNA (Uriu-Adams and Keen 2005).

### Hephaestin

Hephaestin is a homolog of Cp and it shares some of its characteristics. It is transmembrane protein of about 134 kDa, located in trans-Golgi vesicles. It has ferroxidase activity and it is involved in intestinal iron absorption (intestinal iron efflux). It may oxidize iron ions for binding to apotransferrin, allowing its release into the blood as holotransferrin (Tapiero et al. 2003). A consequent of hephaestin deficiency is sex-linked anemia.

### Cartilage matrix glycoprotein

Cartilage matrix glycoprotein—is another intracellular homolog of Cp with ferroxidase and oxidase activities. It is located in the vesicular portions of chondrocytes, as well as in the epithelial cells of the eye. It may play a role in the formation of the extracellular matrix (Tapiero et al. 2003).

### Protein-6-lysine oxidase

Lysyl oxidase plays crucial role in the formation, maturation and stabilization of connective tissues. A lack of this enzyme is manifested by a laxity in collagen and skin and reflects a defect in collagen and elastin crosslinking. Lysyl oxidase is part of the extracellular matrix of organs and tissues in the body, including cartilage and bone. It is a multimer protein composed of 32 kDa subunits, which requires copper for its activity (Tapiero et al. 2003).

Other important Cu-dependent enzymes are: tyrosinase (necessary for melanin production), dopamine-β-hyroxylase (important in catecholamine production), peptidylglycine α-amidating monooxygenase (required for neuropeptide and peptide hormone processing), monoamine oxidase (needed for pigment and neurotransmiter production and metabolism) (Uriu-Adams and Keen 2005).

## Complexes of copper

Current interest in copper complexes comes from their potential use as antimicrobial, antiviral, anti-inflammatory, antitumor agents, enzyme inhibitors or chemical nucleases. Biochemical action of copper’s complexes with non-steroidal anti-inflammatory drugs (NSAIDs) has been recently studied. Numerous Cu(II) complexes of NSAIDs, showing enhanced anti-inflammatory and antiulcerogenic activity as well as reduced gastrointestinal toxicity compared to the un-complexed drug have been prepared and structurally characterized (Joseph and Nagashri 2012). It is likely that copper complexes interact with enzymes and inhibit vital cell functions, rather than interact with DNA and induce crosslinking (Iakovidis et al. 2011). Cu(II) chelates of salicylaldoxime and resoecylaldoxime are potent antiproliferative agents, exhibiting strong cytotoxic effects, comparable to that of adriamycin, by inducing cell arrest and apoptosis (Ferreza et al. 2010). Their action may involve the inhibition of the enzyme topoisomerase II activity by preventing dimer formation of enzyme and its reaction with DNA. The complex 2,6-bis(benzimidazo-2-yl)pyridine copper(II) chloride has been show to exhibit metalloprotease activity. Complexes of carboxyamidrazones exhibit enhanced antiproliferative activity against B16F10 mouse melanoma cells (Fernandes et al. 2012). It is suggested that the combination of copper with carboxyamidrazone ligands may facilitate intracellular transportation and block estrogen receptors. Copper complexes that exhibit high SOD-like activity are potent drugs for prion disease since such activity is correlated with antiprion activity. It is known that prion proteins can bind Cu(II) ions with high specificity as they possess a number of copper sites. Moreover in the development of prion disease, copper may modulate the rate of protein misfolding.

Tetradentate Schiff base ligands derived from Knoevenagel condensation of β-ketoanilides and furfural with O-phenylenediamine and diethylmalonate, and their Cu(II) complexes showed antibacterial activity against *Eschericia coli*, *Salmonella typhi*, *Staphylococcus aureus*, *Kliebsiella pneumoniae* and *Pseudomonas aeruginosa*. Tetradentate Schiff base and their Cu(II) complexes showed antifungial activity against *Aspergillus niger*, *Rhizopus stolonifer*, *Aspergillus flavus*, *Rhizoctonia bataticola* and *Candida albicans*. Antifungal activity of clomiphene citrate copper compounds has been determined against two fungi: *Aspergillus flavus* and *Aspergillus niger* (Creaven et al. 2010). Copper(II) complexes containing Schiff bases derived from S-benzylidene thiocarbamates and saccharinate showed anticancer properties (Katwal et al. 2013). These complexes have been active against the leukemic cell line HL-60 and the activities being higher than the Doxorubicin. Isatin-Schiff base copper(II) complexes possessed pro-apoptotic activity. Copper complex with mixed ligands such as: phenanthroline or 2,2′-bipyridine and acetylacetonate or glycinate are known as cassiopeinas. They exhibit significant antineoplastic activity in vitro and in vivo, against variety of tumor cell lines (Ferreza et al. 2010). Semicarbazone Cu(II) complexes imitate superoxide dismutase activity. Copper(II) complexes with phenolic hydrazone have been used as dyes, bakelite and drugs. Amido-Schiff base form chelates with Cu(II) and act as a thrombin inhibitors. Copper complex with isatin and its derivatives have shown a variety of biological effects, including inhibition of monoamine oxidase. Pyrrolidine dithiocarbamate complexes of Cu(II) were reported to cause the inhibition of the proteasome in vitro against LNCaP prostate cancer cells. These copper complexes resulted in low levels of proteasomal chymotrypsin-like activity and the accumulation of ubiquitinated proteins.
